# Pathological findings in the endometrium after microwave endometrial ablation

**DOI:** 10.1038/s41598-020-77594-x

**Published:** 2020-11-27

**Authors:** Kentaro Nakayama, Sultana Razia, Tomoka Ishibashi, Masako Ishikawa, Hitomi Yamashita, Kohei Nakamura, Kiyoka Sawada, Yuki Yoshimura, Nagisa Tatsumi, Sonomi Kurose, Toshiko Minamoto, Kouji Iida, Noriyoshi Ishikawa, Satoru Kyo

**Affiliations:** 1 Department of Obstetrics and Gynecology, Shimane University School of Medicine, Enyacho 89-1, Izumo, Shimane 6938501 Japan; 2 Department of Organ Pathology, Shimane University School of Medicine, Izumo, 6938501 Japan

**Keywords:** Diseases, Endocrinology, Pathogenesis

## Abstract

The acceptance of MEA in Japan is well demand due to its outstanding effectiveness and safety. Infrequently, a repeat MEA or hysterectomy is needed for recurrent menorrhagia in case of failure ablation. The reasons of recurrent menorrhagia subsequent MEA treatment are unclear. The objective of current study is to identify the possible causes of menorrhagia repetition following MEA, together with the observation of histological changes in the endometrium due to this treatment compared with normal cycling endometrial tissue. A total of 170 patients, 8 (4.7%) of them carried out hysterectomy after 16.8 months (range, 2–29 months) of MEA treatment. Normal (n = 47) and MEA (n = 8) treated paraffin embedded endometrial tissue were prepared for hematoxylin and eosin (H&E) and immunostaining study to recognize the histological changes in the endometrium as a result of MEA treatment. The histological features observed increased tubal metaplasia (TM) including negative expression of the estrogen receptor (ER) and progesterone receptor (PR) in the endometrium subsequent MEA treatment. Increased TM together with the absence of ER and PR expression might be a reasonable explanation for repetition menorrhagia in cases of failure ablation. Further study is required to clarify the molecular mechanisms of tubal metaplasia and the expression loss of hormone receptor in the endometrium as a result of MEA treatment. Current studies propose that low dose estrogen-progestin may not be effective with recurrent menorrhagia patient’s due to the inadequacy of hormone receptor expression in the endometrium following MEA.

## Introduction

Heavy menstrual bleeding (HMB) is a common gynecological problem affecting 20% of women of reproductive age^1, 2^. For clinical purposes, HMB is defined as excessive menstrual blood loss, which interferes with the women’s physical, emotional, and social well-being and quality of life and occurs alone or in combination with other symptoms. A wide range of treatments with varying levels of effectiveness are available for women with HMB. These include drug therapy such as nonsteroidal anti-inflammatory drugs, oral contraceptives, oral progesterone, and hormone-releasing intrauterine device (IUD)^3^. Hysterectomy is the most commonly performed procedure with 100% success rate for HMB treatment when drug therapy is ineffective. However, the complication rate following hysterectomy is up to 40% and mortality rate is 6–11/10,000^4^. Minimally invasive procedures are alternatives to hysterectomy and allow some women to avoid major surgery for HMB. Laparoscopic subtotal hysterectomy (SH), single port access laparoscopy subtotal hysterectomy (SPAL-SH), and hysteroscopic transcervical endometrial resection (TCHE) were introduced as minimally invasive surgical procedures, but did not gain wide acceptance of gynecologic surgeons^5,6^. Microwave endometrial ablation (MEA) is one of the remarkable therapeutic advancements for the treatment of therapy-resistance menorrhagia, and should be considered a standard treatment^7^. MEA was introduced in the Shimane University Department of Obstetrics and Gynecology in August 2007. MEA was authorized as an advanced medical treatment by the ministry of Health, Labor, and Welfare of Japan in June 2009. In April 2012, MEA was approved for national health insurance coverage as K863-3: hysteroscope-assisted endometrial ablation (17,810 points)^7^. Over the past 10 years, we have performed the MEA on more than 200 patients at our institution, and MEA has been proven to be an effective and safe treatment for excessive menstruation with good cost-effectiveness^8,9^. A few reports have described repeat MEA for recurrent menorrhagia following successful MEA treatment^10–12^. The basis of recurrent menorrhagia after MEA is unknown. This study aimed to identify the possible causes of repeat menorrhagia and observe the histological changes in the endometrium following MEA treatment by comparing these findings to those observed in normal cycling endometrial tissue. To the best of our knowledge, no study to date has evaluated the changes in the endometrium after cauterization.

## Results

**Figure 1:**
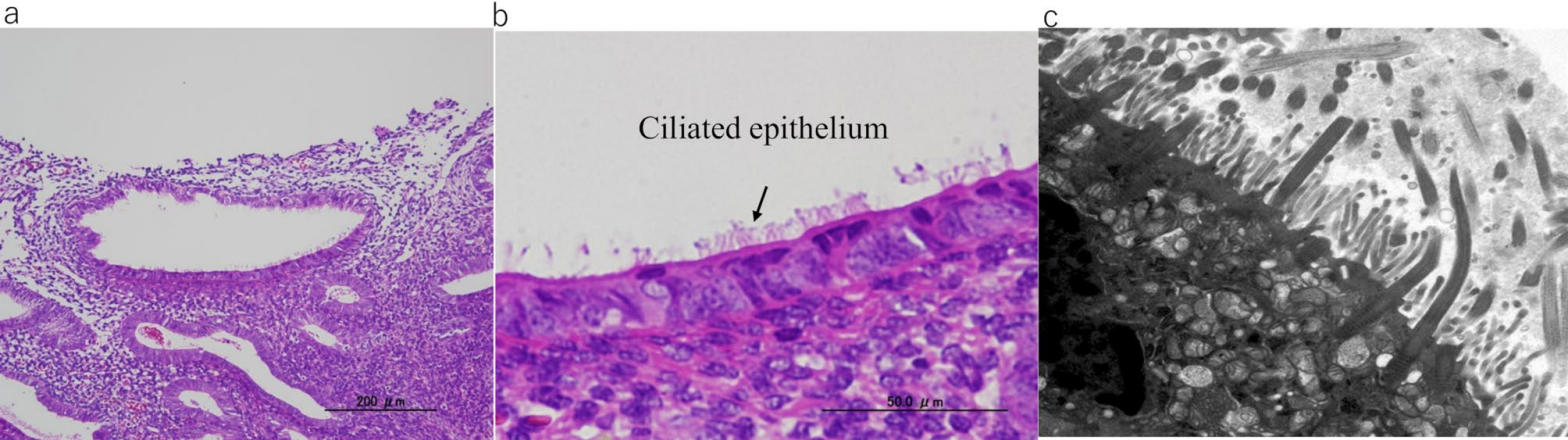
H&E staining of endometrial tissue following MEA treatment (**a**, **b**). (**a**). MEA treatment induced tubal metaplasia in the endometrium that is characterized by the appearance of ciliated cells ( →) (**b**). Electron microscopy image showing obvious cilia in the area of tubal metaplasia after MEA (**c**).

**Figure 2:**
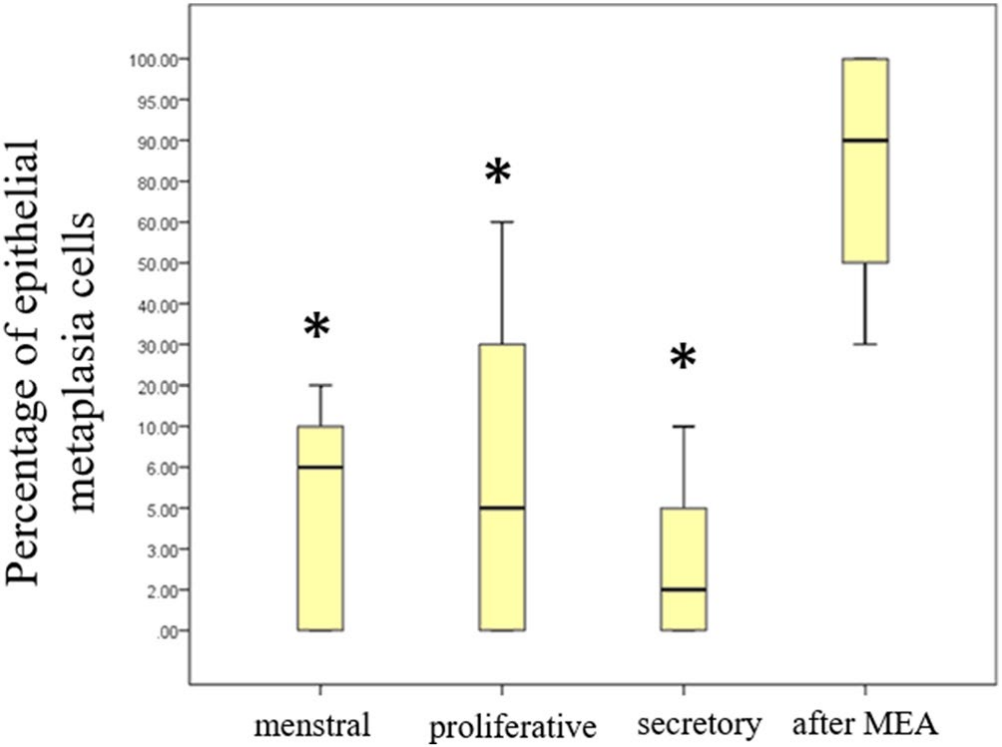
Proportion of tubal metaplasia cells in a normal cycling endometrium compared to that in the endometrium after MEA treatment. The frequency of tubal metaplasia was significantly (*p < 0.05) higher in the endometrium after MEA treatment than in the normal cycling endometrium. The number of cases classified into each group in both the normal cycling endometrium and the endometrium following MEA: menstrual (n = 8), proliferative (n = 8), secretory (n = 8), and after MEA (n = 8).

**Figure 3:**
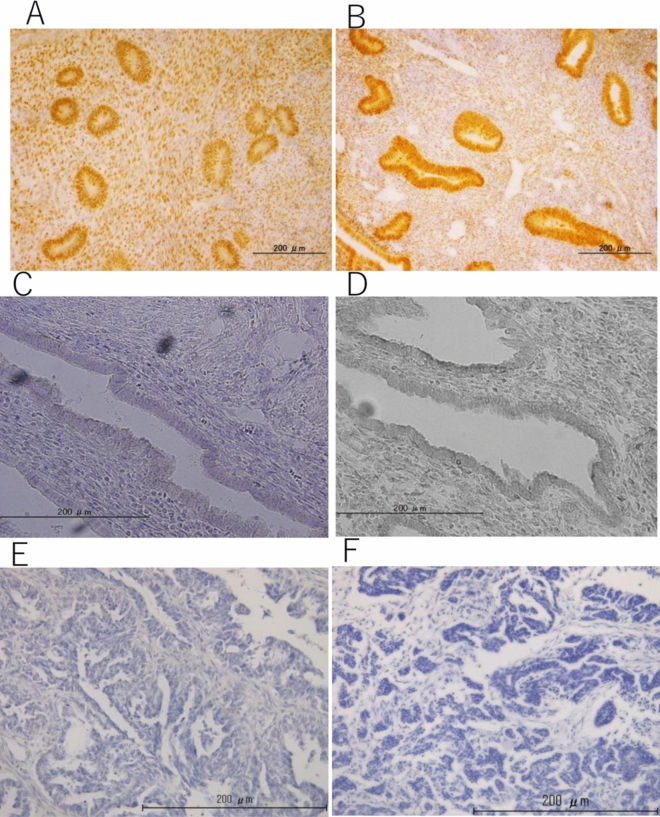
Immunoreactivity of the ER and PR in endometrial tissue. The ER (**A**) and PR (**B**) are expressed in the normal cycling endometrium (early proliferative stage), while the ER (**C**) and PR (**D**) are not expressed in the endometrium subjected to MEA treatment. Ovarian cancer tissue section was used as negative control of ER (**E**) and PR (**F**).

**Figure 4:**
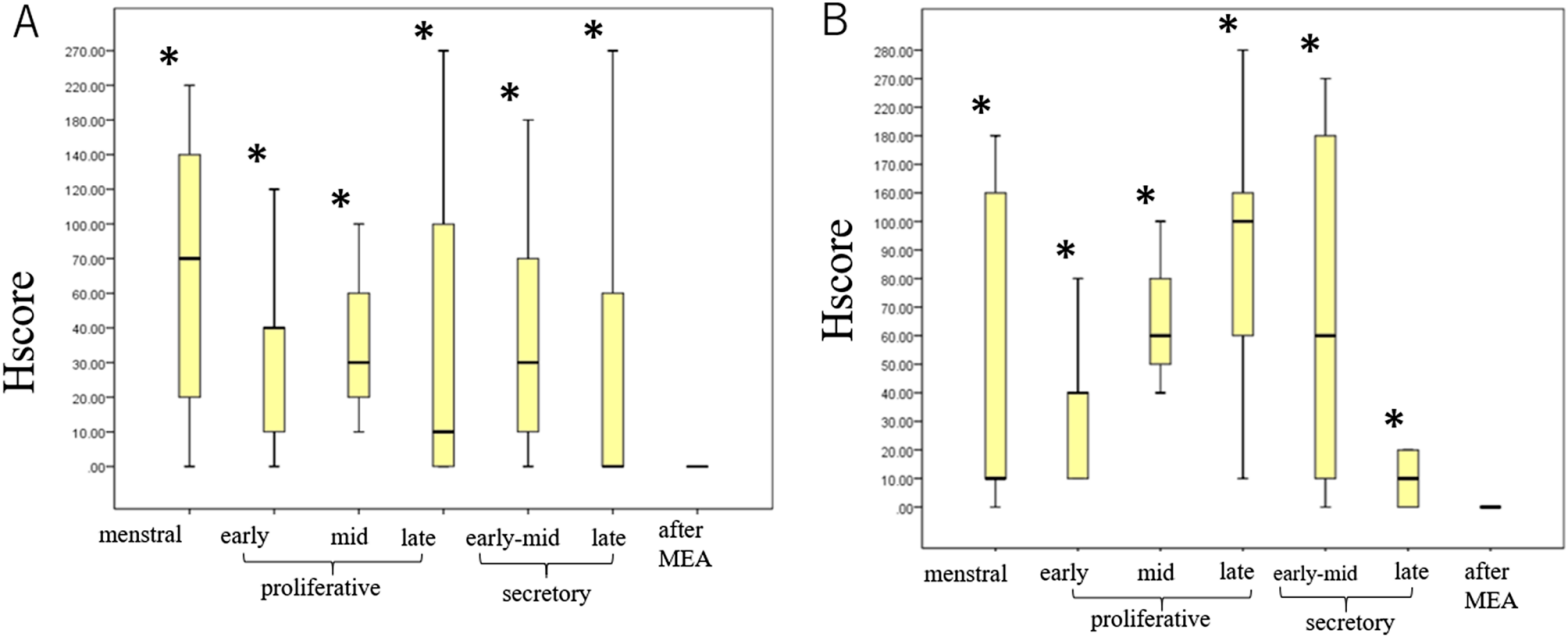
Expression pattern of the ER (**A**) and PR (**B**) in the normal cycling endometrium and endometrium after MEA. The scores (mean ± SD) for the expression of both the ER and PR were significantly (*p < 0.05) decreased in the endometrial tissue after ablation compared to that in the normal cycling endometrial tissue. Cases found in each category in the normal cycling endometrium and endometrium with MEA treatment after immunostaining of hormone receptors: menstrual (n = 5), early proliferative (n = 6), mid proliferative (n = 4), late proliferative (n = 8), early-mid secretory (n = 7), late secretory (n = 17), and after MEA (n = 8).

**Figure 5:**
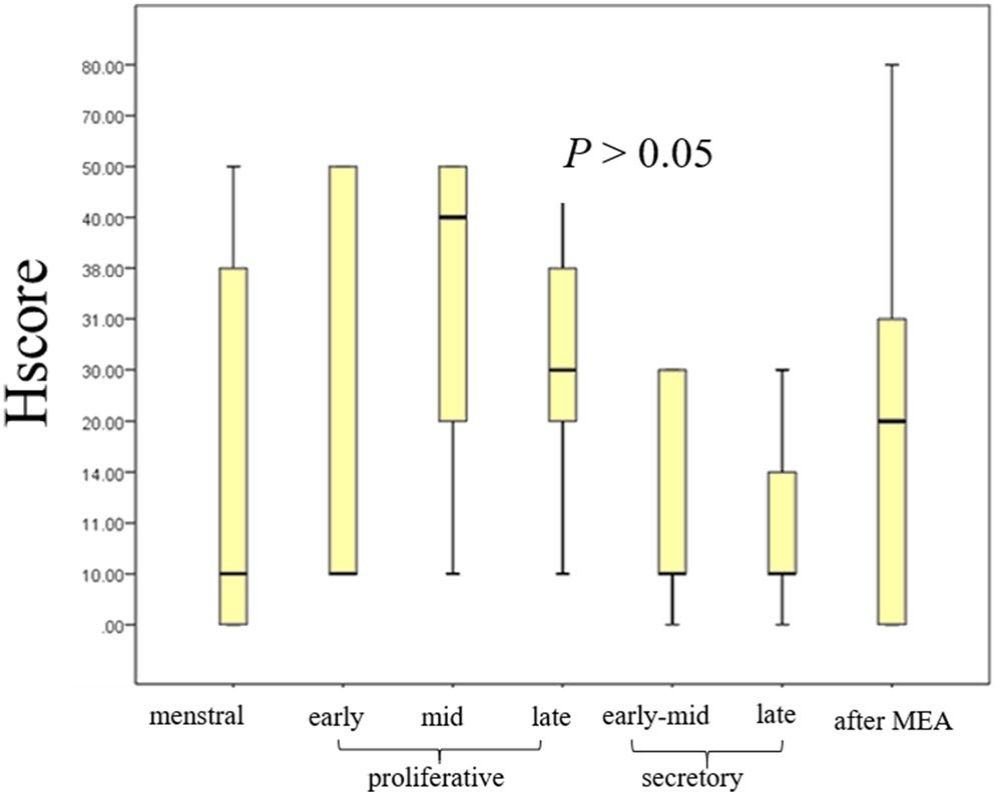
Expression pattern of proliferation marker Ki-67 LI. The scores (mean ± SD) for the expression of Ki-67 LI was not significantly (*p* > 0.05) different between the normal cycling endometrium and the endometrium after MEA treatment. Cases found in each category in the normal cycling endometrium and endometrium with MEA treatment after immunostaining of Ki-67 LI: menstrual (n = 5), early proliferative (n = 6), mid proliferative (n = 4), late proliferative (n = 8), early-mid secretory (n = 7), late secretory (n = 17), and after MEA (n = 8).

Histopathological analysis of tissue obtained from patients after MEA showed endometrial tubal metaplasia (TM), which was characterized by the appearance of ciliated cells, and confirmed by electron microscopy (Fig. 1). TM was observed in all sectioned endometrium that was treated with MEA, and normal endometrial cells were not observed in all sections of the specimens. The frequency of TM in the endometrium after MEA was significantly higher than that in the normal cycling endometrium (*p* < 0.05) (Fig. 2). The expression of hormone receptors was analyzed, and we found that the estrogen receptorα(ER) and progesterone receptor (PR) were not expressed in the endometrium of patients who underwent MEA. However, the ER and PR were adequately expressed in the nuclei of gland cells and endometrial stromal cells of normal cycling endometrium (Fig. 3). Expression of the ER and PR in the gland cells were significantly lower in the endometrium after ablation compared to that in the normal cycling endometrium (*p* < 0.05) (Fig. 4). The proliferation marker Ki-67 labelling index (Ki-67 LI), was adequately expressed in the normal cycling endometrium, as well as in the endometrium after MEA. There were no statistical differences in the expression levels between the 2 groups. (Fig. 5).

## Discussion

MEA is an effective procedure for the management of HMB^8, 9^. The satisfaction rate with MEA is high, ranging from 77–96%^13^, with amenorrhea occurring in 14–70% of patients^14,15^. The quality of life, including pleasure, habit, and discomfort scores, for women with HMB is substantially improved to a normal level 12 months after treatment with MEA^16^. Failure rates of endometrial ablation have been reported to be 5–16%. Failure ablation results hysterectomy or repeat ablation^17–20^. In a study including 67 women who underwent hysterectomy after failed endometrial ablation, bleeding was the most common complaint (51%), followed by pain (28%), and lastly, both bleeding and pain (21%)^11^. Interestingly, in the current study, all patients carried out hysterectomy within 3 years after endometrial ablation. Certainly, the time duration between MEA treatment received and hysterectomy seemed to be short. The persistent hypermenorrhea was significantly associated with subsequent hysterectomy in all patients. However, the reasons of recurrent bleeding after MEA is still unknown.

Knowledge of the histological changes in the endometrium after endometrial ablation could help elucidate the local endometrial mechanisms that contribute to menorrhagia. Therefore, in this study, we compared the histologic findings of normal cycling endometrium and the endometrium after MEA failure. A total of 170 patients, 8 patients underwent hysterectomy because of MEA failure. Clinically, persistent bleeding was the most common reason for having hysterectomy in all patients. Still now, no previous report has been found to describe the histology of the endometrium in women where MEA become failure. We noted increased TM in all the post-ablation endometrial samples. The frequency of TM was significantly higher in the endometrium that treated with MEA than normal cycling. This result suggests that ablation may stimulate the endometrium and facilitate TM.

The expression pattern of the ER and PR in the post-ablation endometrial samples was also compared to that in the normal cycling endometrium. Using immunohistochemical staining, we found that the ER and PR expressions were very low in endometrial samples from patients who received MEA treatment. In contrast, ER and PR were adequately expressed in the epithelial cells in all normal cycling endometrial samples. The scores for both the ER and PR expressions were significantly decreased in the post-ablation endometrial tissue compared to that in the normal cycling endometrial tissue. These results suggest that the endometrium treated with MEA may not undergo hormone-dependent changes. Interestingly, we found that the expression level of Ki67 LI was similar in normal cycling endometrium and the endometrium after MEA, even though the protein expression level of ER and PR were negative in the endometrium that treated with MEA. We speculate that the protein expressions level of ER and PR in the endometrium of MEA were too low to detect by immunohistochemical analysis. ER/PR mRNA expression level might be positive. Our results suggest that lower levels of ER and PR expression might be effective to proliferate the growth of the endometrium. Further study is needed for the measurement of the expression level of ER and PR by q-PCR (RNA levels), as we could not measure due to the lack of frozen tissue samples.

The study findings could not explain the association increased cell metaplasia with MEA treatment. The actual causes of recurrent bleeding after MEA treatment are not known. However, any aberration in the endometrium could hamper its normal development and function. Increased TM and an absence expression of the ER and PR in the endometrium following MEA may impact recurrence.

There are several limitations of our study. First, this study included a small number of patients; therefore, concrete conclusions could not be drawn. Further investigations with larger study populations are, hence, essential. Second, we analyzed surgical outcomes of procedures performed by only one surgeon in our study. This may introduce a bias in our findings. Consequently, further randomized controlled trials are required.

In conclusion, our study revealed that TM and a lack of hormone receptor expression could be responsible for recurrent bleeding following MEA. Thus, low dose estrogen-progestin may not be effective in patients with recurrent bleeding following MEA due to the lack of hormone receptors in the endometrium. Further studies are needed to fully understand the origin and molecular mechanisms of the TM and loss of hormone receptor expression. Research is being conducted in our laboratory to clarify the origin of TM. We are interested in determining whether TM arises from the residual endometrium or from the migration of normal tubal or cervical cells.

## Materials and method

### Study design and data collection

The present study included 200 patients who underwent MEA treatment for menorrhagia at our institution between 2007 and 2014. Out of 200 patients, 170 were followed up as outpatients. Written informed consent for MEA was obtained from all patients. Of the 170 patients, 8 (4.7%) underwent hysterectomy as a result of failure ablation, after 16.8 months (range, 2–29 months) of MEA treatment received. Baseline characteristics of the patients that performed hysterectomy are summarized in Table 1. The mean age of patients was 43.8 (range, 39–47) and 45 (range, 43–50) years at MEA and hysterectomy respectively. Clinical indication for hysterectomy was persistence of abnormal uterine bleeding in all patients. The pathologic diagnoses among the 8 patients were as follows: five patients had uterine fibroids and three had adenomyosis. Endometrial tissues of these 8 patients were used for embedded in paraffin to observe the histological changes following MEA. Normal paraffin embedded endometrial tissues (n = 47) were collected from Shimane University Hospital at the Department of Obstetrics and Gynecology. The patients who only went through a hysterectomy because of non-endometrial gynecologic disorder, were used for tissue samples collection of normal endometria. None of the study participants consumed exogenous hormone prior surgery. Endometrial samples from non-endometrial gynecologic disorder patient were classified into the following 6 groups: early proliferative (n = 6), menstrual (n = 5), mid proliferative (n = 4), early-mid secretory (n = 7), late proliferative (n = 8), and late secretory (n = 17). The specimens were collected after obtaining written consent from all patients with the approval of the Facility Ethical Committee (Shimane University Hospital; approval no. 2004-0381). It was confirmed that all experiments were performed in accordance with relevant guidelines and regulations, such as Helsinki Declaration.

**Table 1:** Characteristics of the patients received hysterectomy after MEA.

Case No	Age at MEA	Age at hysterectomy	Duration untill hysterectomy after MEA (months)	Menstruation after MEA	Reason for hysterectomy after MEA	Parity	Prior Medical Treatment before hysterectomy	LH (mIU/mL)	FSH (mIU/mL)	E2 (pg/mL)
1	43	43	2	Presence	Hypermenorrhea	0	None	6.9	5.8	60
2	47	50	29	Presence	Hypermenorrhea	2	None	3.5	14.7	17
3	46	47	13	Presence	Hypermenorrhea	2	GnRH Analogue 6 months	8.2	2.1	196
4	39	40	10	Presence	Hypermenorrhea	3	None	NA	NA	NA
5	46	48	31	Presence	Hypermenorrhea	2	GnRH Analogue 6 months	14.4	37.7	13
6	43	44	18	Presence	Hypermenorrhea	2	None	NA	NA	NA
7	47	47	3	Presence	Hypermenorrhea	2	None	NA	NA	NA
8	40	43	29	Presence	Hypermenorrhea	3	None	NA	NA	NA

NA: No assessment.

Hormone data (LH, FSH, E2) was analyzed before hysterectomy.

Paraffin embedded tissues (normal and post-MEA) were serially sectioned at a thickness of 5 μm. From each specimen, some sections were stained with hematoxylin and eosin for histologic evaluation and other sections were stained immunohistochemically by indirect immune-peroxidase technique for detection of ER, PR and Ki-67 LI. Immunohistochemistry study was performed on almost the entire uterine lumen of each specimen. Immunohistochemistry was done on deparaffinized sections using ER (DAKO, M7047; Carpinteria, CA), PR (DAKO, M3569; Carpinteria, CA) and Ki-67 LI ( DAKO, M7240; Carpinteria, CA) antibody at a dilution of 1:100. Ovarian cancer tissue section was used as negativo control where as breast cancer cell lysate MCF-7 and T47D were used as positive controls for ER and PR respectively. Sodium citrate buffer was used for antigen retrieval and incubated overnight with each antibody at 4 °C. The slides were examined by two investigators and was scored as follows: 0, undetectable; 1, weakly positive; 2, moderately positive; and 3, intensely positive.

For TEM, the specimens were fixed by immersing them into a mixture of 2.5% glutaraldehyde and 2% paraformaldehyde in 0.1 M phosphate buffer, pH 7.3. They were then fixed with buffered 1% OsO_4_ for 2 h. After dehydration, the specimens were embedded in Epoxy resin type TAAB 812 and stained with 4% uranyl acetate and lead citrate. They were examined with a JEOL JEM 1200EX (JOEL, Osaka, Japan) at 80 kV. The slides were evaluated for all areas of the uterine endometrium quantitatively by three investigators (KN, SR, TI).

### Statistical analyses

Data among the groups were compared using Kruskal–Wallis test followed by Mann–Whitney U test. *P*-values less than 0.05 were considered statistically significant.

### Ethical approval

The acquisition of tumor tissues was approved by the Shimane University Institutional Review Board (IRB No. 2004-0381).

### Informed consent

After appropriate explanation, patients who could provide written informed consent for the procedure and for participation in the study were enrolled. For those patients who were unable to re-visit the hospitals, we clearly announced the opportunity to opt out through measures. Such measures included the addition of information regarding opting out on the hospital website, as well as by putting arrangements in place so that the patients can opt out via the website at any time.

## Abbreviations

MEA: Microwave endometrial ablation
HMB: Heavy menstrual bleeding
TM: Tubal metaplasia
PR: Progesterone receptor
ER: Estrogen receptor
